# Biochelates from Spent Coffee Grounds Increases Iron Levels in Dutch Cucumbers but Affects Their Antioxidant Capacity

**DOI:** 10.3390/antiox13040465

**Published:** 2024-04-15

**Authors:** Beatriz Navajas-Porras, Ana Cervera-Mata, Alejandro Fernández-Arteaga, Adriana Delgado-Osorio, Miguel Navarro-Moreno, Daniel Hinojosa-Nogueira, Silvia Pastoriza, Gabriel Delgado, Miguel Navarro-Alarcón, José Ángel Rufián-Henares

**Affiliations:** 1 Departamento de Nutrición y Bromatología, Instituto de Nutrición y Tecnología de Alimentos, Centro de Investigación Biomédica, Universidad de Granada, 18071 Granada, Spain; beatriznavajas@ugr.es (B.N.-P.); adrianadelgado@ugr.es (A.D.-O.); miguelnav@correo.ugr.es (M.N.-M.); dhinojosa@ugr.es (D.H.-N.); spdelacueva@ugr.es (S.P.); nalarcon@ugr.es (M.N.-A.); 2 Department of Soil Science and Agricultural Chemistry, Faculty of Pharmacy, University of Granada, 18071 Granada, Spain; anacervera@ugr.es (A.C.-M.); gdelgado@ugr.es (G.D.); 3 Department of Chemical Engineering, University of Granada, 18071 Granada, Spain; jandro@ugr.es; 4 Instituto de Investigación Biosanitaria Ibs.Granada, Universidad de Granada, 18012 Granada, Spain

**Keywords:** cucumber, iron, spent coffee grounds, *in vitro* digestion/fermentation, antioxidant capacity, short-chain fatty acids

## Abstract

Spent coffee grounds (SCG) are a type of food waste and are produced in abundance around the world. However, their utilization as a soil organic amendment is challenging due to their phytotoxic effect. In the present work, the impact of agronomic biofortification on Dutch cucumbers was investigated using different chemically modified SCG and analyzing their effects on iron contents, their capacity for releasing antioxidants, and the production of short-chain fatty acids after *in vitro* digestion–fermentation. The results indicated variations in the iron contents and chemical compositions of cucumbers according to the treatment groups. Functionalized and activated hydrochar from SCG increased Fe levels in cucumbers. Although activated hydrochar obtained at 160 °C and functionalized with Fe showed the highest iron supply per serving, differences in antioxidant capacity and short-chain fatty acid production were observed between the groups. It is concluded that growing conditions and the presence of iron may significantly influence the contribution of these cucumbers to the dietary intake of nutrients and antioxidants, which could have important implications for human health and nutrition.

## 1. Introduction

Spent coffee grounds (SCG) are the main by-products obtained during coffee brewing, mainly in coffee shops, with a worldwide production of around 15 million tons/year [1]. SCG have a positive impact on soil quality [2], so they can improve crop growth in the short term by increasing organic matter levels, soil fertility, and carbon stock [3]. However, some compounds present in the dregs can be toxic and can reduce microbial diversity when they accumulate, so they should be used in moderation [2]. An alternative substance is biochar, which is stable carbonaceous material that can remain in soil for hundreds of years, improving crop fertility and plant growth [4]. It also has environmental benefits, as it can reduce greenhouse gas emissions and promote sustainable management of organic waste [5]. Hydrochar is similar to biochar but is produced via hydrolysis instead of pyrolysis [6].

To improve the physical, chemical, and biological properties of crops and soil quality, organic amendment (the addition of organic matter to croplands) is carried out. This practice is commonly used owing to its positive influence on soil biodiversity, biochemical parameters, and provision of essential nutrients for crop growth, among other benefits [7]. However, organic amendment has some disadvantages, as the quality and composition of amendments may vary, being limited in certain regions, or nutrient supply may be slower than with other fertilizers [8]. Accordingly, alternatives to organic matter have been proposed for use in crop improvement.

In the context of organic amendment, agronomic biofortification is a procedure that aims to increase the contents of essential nutrients in crops and to combat malnutrition in populations that depend on crops as their main food source [9]. Biofortification can increase the mineral content in the edible portion of a crop, thereby improving the nutritional value of food. In addition, micronutrient fertilization can have a positive impact on other nutritional parameters of crops, such as protein content, amino acids, phenolic compounds, chlorophyll, carotenoids, and essential oils [10]. One of the most commonly used elements in agronomic biofortification is Fe, as it is an essential mineral for humans as well as plants [9,11].

Cucumber (*Cucumis sativus* L.) belongs to the Cucurbitaceae family and is one of the most cultivated vegetables in the world. Despite its high water content, it contains bioactive compounds that provide remarkable antioxidant capacities, such as tannins, terpenoids, saponins, cardiac glycosides, and dietary fiber [12]. Dietary fiber (and attached phenolic compounds) is poorly digested by humans and is transformed by the gut microbiota in the colon into simpler metabolites with enhanced absorption and bioactivity [13]. The main metabolites of dietary fiber are short-chain fatty acids (SCFAs), such as acetic, propionic, and butyric acids, which have proven health properties [14]. In addition, gut microbiota also releases antioxidant compounds from the food matrix [15].

Taking all of the above-mentioned information into account, the aim of this study was to evaluate the effect of agronomic biofortification of Dutch cucumber using different soil treatments based on SCG: activated spent coffee grounds (ASCG), Fe-functionalized and activated coffee grounds (ASCG-Fe), activated hydrochar obtained from SCG at 160 °C (AH160), and Fe-functionalized and activated SCG hydrochar (AH160-Fe). Changes in iron contents and the release of antioxidants and production of SCFAs after simulated human digestion–fermentation were assessed. Finally, the contribution of the consumption of cucumbers to the daily intake of iron and polyphenols in the Spanish diet was calculated. It is important to highlight that mineral biofortification is an innovative and relevant strategy due to the importance of minerals in health, the prevalence of mineral deficiencies in the diet, and its potential as a sustainable agronomic solution [10]. Thus, the novelty of this paper compared with the existing literature relies on three aspects: (i) the use of a food waste (spent coffee grounds) as a source of biochelates for mineral biofortification; (ii) the study of the effects of Fe biofortification on other nutritional parameters (such as total phenolic content and the release of antioxidants and short-chain fatty acids after *in vitro* digestion–fermentation); and (iii) evaluation of the contribution of the biofortified cucumbers on daily Fe and polyphenol intakes.

## 2. Materials and Methods

### 2.1. SCG BioProducts

SCG were acquired from the cafeteria of the Faculty of Pharmacy (University of Granada). They were spread into a thin layer and dried at room temperature (18–21 °C) for 7 days to remove residual moisture. Four soil treatments (based on SCG) were obtained applying the methodology described by Lara-Ramos et al. [16]: SCG hydrochar at 160 °C (H160), activated SCG (ASCG) and hydrochar (AH160), and their corresponding activated and functionalized Fe biochelates (ASCG-Fe, AH160-Fe). The Fe contents of the biochelates were determined by mineralization with HNO_3_ (65% *v*/*v*) and H_2_O_2_ (30% *v*/*v*) at 185 °C for 20 min in a microwave digestor (Multiwave 5000 with Rotor 24HVT50, Anton Paar GmbH, Graz, Austria). Mineral ions in the extracts were measured using inductively coupled plasma mass spectrometry (ICP-MS/MS; Agilent 8900 Triple Quadrupole ICP-MS/MS, Agilent Technologies Inc., Santa Clara, CA, USA). The final Fe contents of the bioproducts used in this study, expressed in mg/kg, were: ASCG = 59.3 ± 1.2; AH160 = 60.2 ± 3.2; ASCG-Fe = 12,083 ± 129; AH160-Fe = 13,103 ± 316.

### 2.2. Greenhouse Experiment

The experiment was conducted in a greenhouse at the “Fundación Cajamar” Experimental Station located in Almería, Spain (36°47′23″ N, 2°43′13″ W; 155 m a.s.l.) from October 2022 to January 2023. The site has a semi-arid subtropical Mediterranean climate with an average minimum temperature of 12.2 °C and an average maximum temperature of 16.1 °C; with these oscillations in external temperature, the mean temperature in the greenhouse was 23.0 °C (ranging from 19.2 to 25.6 °C). The average annual precipitation is 220.5 mm, and the mean photosynthetically active radiation is 35.7 E/m^2^. The plot used was 30 m long and 8.5 m wide, with a total usable area of 255 m^2^.

The soil used in the experiment was previously characterized by the staff of the experimental station. According to the internal report, the soil has the following properties: sand 69.79%, silt 17.57%, clay 12.64%, pH 7.4, electrical conductivity at 25 °C (EC25) 2.05 dS/m, organic carbon (OC) 1.35%, carbonates as CaCO_3_ 29.2%, C/N 8.06, total N 0.17%, and available P 318.1 mg/kg.

The assayed bioproducts at doses of 0.2% were: ASCG, AH160, ASCG-Fe, and AH160-Fe. These bioproducts were added at 0.2%, as this is the required dosage to avoid phytotoxicity in plants [16]. The 0.2% dosage corresponds to different amounts of Fe added (mg/kg) with the biochelates; i.e., ASCG: 0.104; AH160: 0.105; ASCG-Fe: 21; and AH160-Fe: 23. Two controls were established: soil without any bioproducts (Control), and a commercial Fe chelate at a concentration of 10 mg/kg soil (Control-Fe). The commercial chelate iron ethylenediamine-N,N′-bis (EDDHA-Fe, 6% *w*/*w*) was supplied by Trade Corporation International, S.A.U. The study involved a total of six treatments (Table 1). The experiment was set up with four replicates in a split-plot design. Two plots were used, each containing one replicate per treatment and composed of four plants distributed at random. Overall, each treatment consisted of eight plants (*n* = 8) per plot.

The planting points were distributed as follows: 1.5 m between crop lines and 0.5 m between plants within each crop line, equating to a planting density of 1.33 plants/m^2^. For the placement of the bioproducts, a 20 cm deep × 15 cm diameter hole, equivalent to 4 kg of soil, was made at each planting point. The soil in all holes corresponding to the same treatment was extracted and homogenized for 5 min with the corresponding bioproduct in a concrete mixer. The holes were then individually re-filled with the soil–bioproduct mixture. The holes of the controls had the same soil treatment. The commercial chelate was dissolved in distilled water and poured on the surface.

Cucumber seedlings (*Cucumis sativus* L. var. “Almería” cv. “Huracán”) of 21 days old were acquired from the commercial greenhouse Acrena S.A.T 251 (Almería, Spain). One seedling per point was planted at a soil depth of 8 cm 6 days after the soil preparation.

### 2.3. Crop Maintenance

The experiment was managed organically, using fertilizers authorized for organic cultivation by applying the drip irrigation method. Each row of cucumber was irrigated using a polyethylene pipe. The emitters were spaced at 50 cm intervals with an irrigation rate of 3 L/(h·m^2^). After the first 15 days of the transplant, the cultivation was irrigated with a nutrient solution containing 10 mM/L of N, 1.41 mM/L of P, and 5.56 mM/L of K based on an organic fertilizer with a richness of 3.5% N, 2.5% P_2_O_5_, and 6.5% K_2_O diluted to a concentration of 3.2‰. The concentrations of nitrate, potassium, calcium, and sodium in the extracted soil solution were monitored biweekly using suction probes to maintain a nitrate concentration in the range of 3 to 10 mM/Land a K/Ca ratio between 0.5 and 1. These concentrations were measured used rapid analysis ionometers (LAQUAtwin, Horiba, Kyoto, Japan).

### 2.4. Plant Sampling and Processing

Cucumbers were harvested after 105 days of cultivation. Then, the fruits were cleaned using drinking water, chopped, and divided for analysis of the antioxidant capacity of Fe. Samples for antioxidant capacity analysis were stored at −80 °C until *in vitro* digestion–fermentation. The samples for Fe analysis were dried in aluminum trays at room temperature for 48 h, then placed in an oven at 50 °C for 72 h and weighed again (dry weight). The dried material was milled (Model-150, Lejieyin, China) and stored at room temperature until analysis.

### 2.5. Fe Determination

For the determination of Fe contents in the studied samples, 0.200 g of the homogenized and dried sample was weighed using a precision balance (Ohaus, model PA224C, Europe GmbH, Greifensee, Switzerland) inside borosilicate tubes, and 3 mL of 69% HNO_3_ (TraceSELECT, Honeywell, Fluka, France) was added. Next, 3 mL of an 11.5% HNO_3_ solution was added to the Teflon cups of the microwave digester, in which the borosilicate tubes with the samples to be mineralized were placed. The Teflon digestion vessels were then placed in the rotor of the microwave digester and, after optimization of an appropriate time–temperature program (30 min for a temperature range from 150 to 180 °C), the samples were mineralized.

After mineralization, the samples were diluted to 50 mL with Milli-Q reagent-grade water to obtain the analytical solution, with an acidity of 4.14% HNO_3_, in which the measurement of Fe was carried out using ICP-MS/MS.

For the final determination of Fe, a calibration curve was prepared from the standard solution of Fe of 1000 mg/L in HNO_3_ at 1% (ppm; Merck; Darmstadt, Germany). The stock solution used was 100 mg/L (ppm), from which the different points of the linear calibration curve were prepared using serial dilutions (1, 10, 25, 50, 500, and 1000 ng/L, ppb). The internal standard used in the measurement was the “Internal Standard Kit (Ge, Ir, Rh, Sc; ISC Science, batch 20210712)”, which was used for correction of the counts per second (CPS) of Fe for its analyzed atomic mass (^54^Fe), using that of the mineral of the internal standard ^72^Ge. The measurements were carried out in triplicate for each of the analyzed samples.

Before measuring Fe concentrations in cucumber samples, we studied the analytical parameters of the method used. The limit of detection (LOD) was 0.11 μg/L. In the study of the accuracy and precision of the method (*n* = 10), the Fe-certified reference standards “Bovine muscle powder No. 8414” and “Citrus leaves powder No. 1515” certified by the National Institute for Standards and Technology (NIST; Gaithersburg, MD, USA) yielded concentrations of 70.5 ± 1.9 and 82.7 ± 2.60 μg/g for certified levels of 71.2 ± 9.2 and 83.1 ± 3.00, respectively, with no statistically significant differences (*p* > 0.05). With regard to the recovery percentage, after the addition of Fe to the samples and prior to their preparation [5], values of Fe ranging from 97.8 to 101.6% were obtained. These results and those corresponding to the coefficients of variation obtained with an average value of 2.83% show the suitability of the mineralization and analysis technique used for the measurement of Fe in cucumber samples.

To evaluate the efficiency of the different bioproducts used, the Fe utilization efficiency (UE) was calculated according to Zhao et al. [17]:

UE (%) = (Uptake in treatment-uptake in control)/Micronutrient added × 100.

### 2.6. In Vitro Digestion and Fermentation

Samples were subjected to *in vitro* gastrointestinal fermentation and *in vitro* fermentation in triplicate, following previously described protocols [18]. First, an oral phase was carried out, in which cucumbers were added to falcon tubes with simulated salivary fluid (1:1, *w*/*v*) composed of salts and α-amylase (75 U/mL). The falcon tubes were held for 2 min at 37 °C under oscillation.

Then, the gastric phase was performed. For this, 5 mL of simulated gastric fluid was added, simulating the salts and pepsin contents of gastric juices (2000 U/mL). The mixture was held at 37 °C for 120 min at pH 3 and in oscillation. Finally, the intestinal phase was carried out, in which 10 mL of simulated intestinal fluid was added, simulating the content of intestinal juices in salts, bile salts, and enzymes (67.2 mg/mL pancreatin was used). The mixture was held at 37 °C for 120 min in oscillation, as in the gastric phase, but this time at pH 7. Once the intestinal phase was finished, the tubes were placed on ice and the enzymatic reactions were stopped. The tubes were then centrifuged for 10 min at 16,200× *g*. The resulting solid pellet served as an *in vitro* fermentation substrate and represents the undigested portion entering the large intestine. The supernatant, which represents the fraction available for absorption in the small intestine, was stored in 1 mL tubes at −80 °C until analysis.

*In vitro* fermentation was carried out using fecal samples from five healthy donors with an average Body Mass Index = 21.3 who had not taken antibiotics during the three months prior to the trial. The feces were pulled to reduce inter-individual variability. The fermentation was carried out for 20 h at 37 °C in oscillation. After completion of the *in vitro* fermentation, the samples were placed on ice, as after digestion, to stop microbial reactions and then centrifuged at 16,200× *g* for 10 min. The supernatant, representing the fraction available for absorption in the large intestine, was stored at −80 °C until analysis. The solid pellet, representing the portion that was not fermented, was properly disposed of.

The *in vitro* gastrointestinal digestion and fermentation resulted in the production of two fractions: the digestion supernatant, which corresponds to absorption in the small intestine, and the fermentation supernatant, which corresponds to absorption in the large intestine.

### 2.7. Antioxidant Tests

The antioxidant capacities of the supernatants obtained after digestion and fermentation were studied; the sum of both was considered the total antioxidant capacity [19].

For each antioxidant method and for the determination of polyphenols, samples were prepared in the same way. The supernatants of the *in vitro* digestion and fermentation were taken; then, several dilutions were carried out (as the absorbance of the sample has to enter the calibration curve). The different dilutions were injected into 96-well plates, and the corresponding reagents were added later.

#### 2.7.1. Trolox Equivalent Antioxidant Capacity Versus Reducing Capacity (TEAC_FRAP_ Assay)

The procedure used to determine the ability of the samples to reduce ferric iron was based on that described by Benzie & Strain [20] and modified for use with a microplate reader (Cytation 5, Agilent Technologies Inc., Santa Clara, CA, USA). In brief, 280 μL of freshly prepared FRAP reagent was combined with 20 μL of digestion or fermentation supernatant in a 96-well plate. The antioxidant response was observed for half an hour. Trolox was used to create a calibration curve at a concentration of 0.01 to 4 mg/mL. The results were expressed using mmol Trolox equivalent/kg cucumber fresh weight.

#### 2.7.2. Trolox Equivalent Antioxidant Capacity Versus DPPH Radicals (TEAC_DPPH_ Assay)

The technique was modified to work with a microplate reader (Cytation 5, Agilent Technologies Inc., Santa Clara, CA, USA) and followed the protocol of Yen & Chen [21]. In brief, 280 μL of DPPH reagent was combined with 20 μL of digestion supernatant or fermentation supernatant in duplicate. To make the reagent, 7.4 mg DPPH per 100 mL methanol was used. The antioxidant response was observed for a full hour. Trolox was used to create a calibration curve, with concentrations ranging from 0.01 to 4 mg/mL. Results were given in mmol Trolox equivalent/kg cucumber fresh weight.

#### 2.7.3. Trolox Equivalent Antioxidant Capacity Versus ABTS^+^ Radicals (TEAC_ABTS_ Assay)

The ABTS assay was conducted as described by Re et al. [22] with slight modifications. A stock solution of 7 mM ABTS was combined with 2.45 mM potassium persulphate to create ABTS+, which was then allowed to stand at room temperature for 12–16 h in the dark before use. The ABTS working solution, which is stable for two days, was diluted with ethanol:water (50:50) to an absorbance of 0.70 ± 0.02 at 730 nm. After 20 min, the absorbance was assessed using a Cytation 5 microplate reader (Agilent Technologies Inc., Santa Clara, CA, USA). Trolox dilutions (0.15–1.15 mM) were used for calibration. Results were given in mmol Trolox equivalent/kg cucumber fresh weight.

#### 2.7.4. Total Phenolic Content: Folin–Ciocalteu (FC) Assay

A microplate reader (Cytation 5, Agilent Technologies Inc., Santa Clara, CA, USA) was used to estimate the total phenolic content. The procedure of Singleton & Rossi [23] was followed, with some modifications. For the Folin–Ciocalteu method, the sample, two different reagents (Folin–Ciocalteu reagent and 10% *w*/*v* sodium carbonate), and 190 μL of distilled water must be injected into each plate well. The Folin–Ciocalteu assay was developed in such a way that the water and one of the two reagents are together in the same Falcon tube, and only two different solutions have to be injected. The results were expressed as mg gallic acid equivalents (GAE) per kg of cucumber fresh weight.

### 2.8. Analysis of Short-Chain Fatty Acids

Following a previous study [24], analysis of the SCFAs was performed using ultra-high performance liquid chromatography (UHPLC). There was no need for sample pre-treatment prior to injection. SCFA standards of acetic, butyric, lactic, propionic, and succinic acids (all from Merck; Darmstadt, Germany), prepared in the mobile phase at concentrations ranging from 5 to 10,000 ppm, were quickly created. The mobile phase was supplied at a flow rate of 0.250 mL/min and consisted of a mixture of two solutions. The mobile phase consisted of aqueous acetonitrile (1%) and ultrapure water (99%), both acidified with 1% formic acid. After fermentation, 1 mL of the supernatant was centrifuged, filtered through a 0.22 µm nylon filter, and then transferred to a vial for UHPLC analysis. The UV–Vis photodiode array detector (PDA) was set at 210 nm, and the column was a reversed-phase Accucore™ C18 (ThermoFisher Scientific, Waltham, MA, USA) with a particle size of 2.6 µm and length of 150 mm set at 35 °C. The analysis was performed twice, and the information shown represents the mean values of the millimolar (mM) concentration of each SCFA.

### 2.9. Calculation of Daily Antioxidant and Fe Intakes

The individual contribution of each cucumber group, depending on treatment and plots, to the total dietary intake of polyphenols and Fe was calculated. This took into account (i) the daily consumption of cucumber in Spain and the amount of cucumber per portion [25]; (ii) the total phenolic content previously measured for the samples after the FC test; and (iii) the Fe content of each cucumber group. The total phenolic and Fe contents of each food were adjusted to the usual serving in Spain [26] and compared with previously published results for polyphenols [27] and Fe intake [28].

### 2.10. Statistical Analysis

First, the normality of the samples was studied using the Shapiro–Wilk test. Then, a one-way analysis of variance (ANOVA) was performed for statistical differences between treatments, which were assessed using Tukey’s test. Student’s *t*-test was also performed for graphical plots. The significance level was set at 95% (*p* < 0.05). Statistical analysis was carried out in triplicate and performed using control (non-bioproduct) cucumbers as the reference group, as well as comparisons among all cucumber groups with different treatments. Statgraphics Plus, version 5.1, and SPSS 26.0 for Windows (IBM SPSS Inc., New York, NY, USA) were used.

## 3. Results

### 3.1. Fe Content

**Figure 1:**
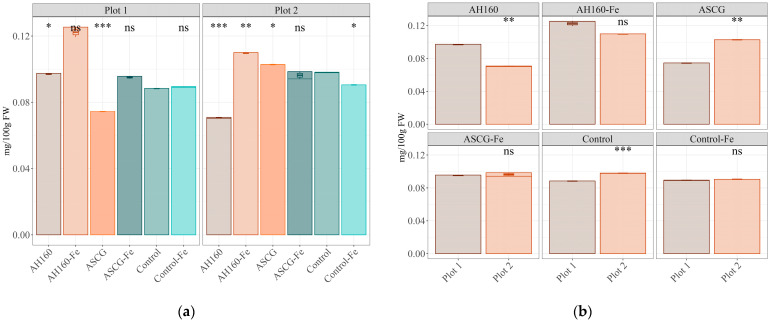
Differences in the Fe contents of cucumbers. (**a**) Differences among treatment groups. Statistical analysis was performed using Student’s *t*-test with the control group as the reference group. Statistical labels: *: *p* < 0.05, **: *p* < 0.01, ***: *p* < 0.001, ns: not significant. (**b**) Differences between plots for each group. Statistical analysis was performed using Student’s *t*-test with Plot 1 as the reference group. Statistical labels: **: *p* < 0.01, ***: *p* < 0.001, ns: not significant.

The Fe contents for each group of cucumbers showed some statistically significant differences (*p* < 0.05) in both plots (Figure 1a,b). First, comparisons were made with the control group (Figure 1a). In cucumbers from plot 1, the AH160 group had more iron (*p* < 0.05) than those of the control group. The treatment that induced the greatest increase in iron contents in the cucumbers was AH160-Fe, reaching levels of 0.122 mg/100 g of fresh weight; however, this increase was not significant due to the large variation in Fe contents between the samples of the AH160-Fe group. The opposite was found for cucumbers of the ASCG group, which had lower iron contents (*p* < 0.001) than the control group. When comparisons were made between the different groups (Table 2), in addition to those already mentioned with the control group, statistically significant differences (*p* < 0.05) were found between all groups except between the pairs ASCG-Fe vs. AH160 and Control-Fe vs. ASCG-Fe. See Supplemental Table S1.

In the case of plot 2, cucumbers from the AH160 and control-Fe groups had lower iron contents (*p* < 0.05) than those from the control group. The opposite trend was found for cucumbers from the AH160-Fe and ASCG groups, which had higher iron levels (*p* < 0.05) than the control group (0.110 and 0.103 mg/100g fresh weight, respectively). When comparisons were made between the different groups (Table 2), statistically significant differences (*p* < 0.05) were found between all groups except between the control-Fe and ASCG-Fe groups.

Finally, differences between the two plots were studied for each cucumber group (Figure 1b). Statistically significant differences were found for the AH160, ASCG, and control groups. Cucumbers from plot 2 had more iron (*p* < 0.05) than those from plot 1 for the ASCG and control groups, whereas the opposite was found for the AH160 group.

### 3.2. Antioxidant Capacity

**Figure 2:**
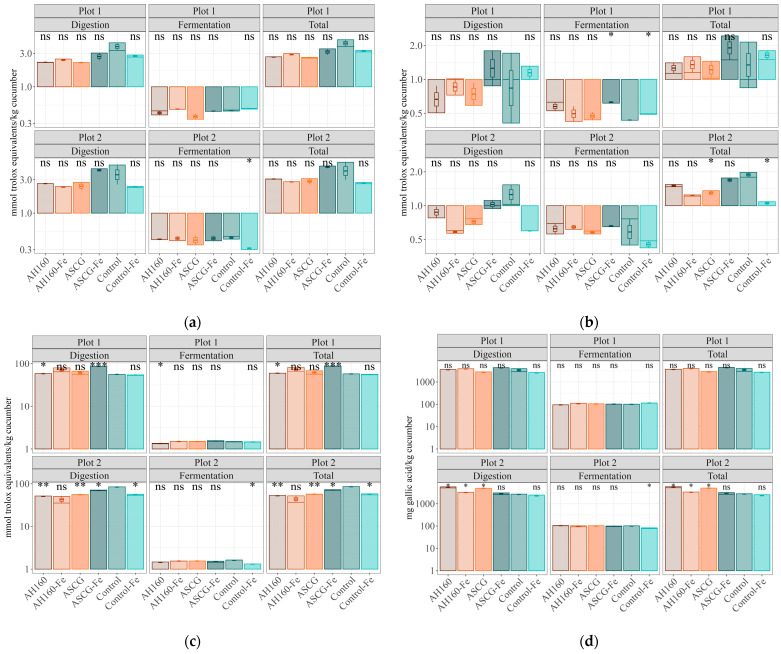
Antioxidant capacities of the different cucumber groups (obtained after *in vitro* digestion, *in vitro* fermentation, and total antioxidant capacity assessment) calculated for fresh weight. (**a**) The Trolox equivalent antioxidant capacity versus reducing capacity (TEAC_FRAP_). (**b**) The Trolox equivalent antioxidant capacity versus DPPH radicals (TEAC_DPPH_). (**c**) The Trolox equivalent antioxidant capacity versus ABTS radicals (TEAC_ABTS_). (**d**) Total phenolic content (Folin–Ciocalteu). Results are log10 transformed to improve visualization. Statistical analysis was performed using Student’s *t*-test with the control group as the reference group. Statistical labels: *: *p* < 0.05, **: *p* < 0.01, ***: *p* < 0.001, ns: not significant.

With regard to the antioxidant capacity released after *in vitro* digestion and fermentation, comparisons were made with those cucumbers belonging to the control group. For the FRAP method, no statistically significant differences were found in plot 1 (Figure 2a). In plot 2, only the antioxidant capacity of the control-Fe group was significantly lower (*p* < 0.05) than that of the control group in the *in vitro* fermentation fraction. In addition, comparisons were made between all cucumber groups, taking into account the total antioxidant capacity (See Supplemental Table S1). Statistically significant differences were not found between the groups in either of the two plots (Table 2).

In the case of the DPPH method (Figure 2b), in which all groups were compared with the control, statistically significant differences (*p* < 0.05) were found in the *in vitro* fermentation fraction for samples from plot 1, where ASCG-Fe and control-Fe cucumbers showed less antioxidant capacity than those of the control group. In plot 2, statistically significant differences were found for total antioxidant capacity, where ASCG-Fe and control-Fe cucumbers had significantly (*p* < 0.05) lower antioxidant capacity than the control group. When studying the comparisons between the different groups (Table 2), taking into account the total antioxidant capacity, for plot 1 there were no significant differences. However, for plot 2, more differences were found in addition to those already mentioned. The antioxidant capacity of the control-Fe group was statistically significantly lower (*p* < 0.05) compared with the AH160 and ASCG-Fe groups. In addition, the antioxidant capacity of the ASCG-Fe group was higher compared with the AH160-Fe and ASCG groups.

For the ABTS method, when comparisons were made with the control group (Figure 2c), significant differences were found for both fractions (*in vitro* digestion, *in vitro* fermentation) and total antioxidant capacity. In plot 1, statistically significant differences (*p* < 0.05) were obtained between the AH160 cucumbers and the control group, as well as between ASCG-Fe and the control group (in the digestion fraction and total antioxidant capacity); the antioxidant capacity of the AH160 and ASCG-Fe groups was higher. However, in the *in vitro* fermentation fraction, the antioxidant capacity of the AH160 group was significantly (*p* < 0.05) lower than that of the control group. For the samples from plot 2, the antioxidant capacities of the AH160, ASCG, ASCG-Fe, and control-Fe groups were lower (*p* < 0.05) than that of the control group (for the *in vitro* digestion fraction and total antioxidant capacity). In the *in vitro* fermentation fraction, only the antioxidant capacity of the control-Fe group was significantly lower (*p* < 0.05) than that of the control group. When multiple comparisons (ANOVA) were performed among all cucumber groups (Table 2), statistically significant differences were also found. In addition to those already stated for plot 1 with respect to the control group, it was found that the antioxidant capacity of ASCG-Fe cucumbers was significantly higher than those of the AH160, ASCG, and control-Fe groups (*p* < 0.05). For plot 2, the antioxidant capacity of the ASCG-Fe group was again higher than those of the AH16 and AH160-Fe groups (*p* < 0.05).

Finally, for the FC method, no significant differences were found in plot 1 (Figure 2d) when comparisons were made with the control group. In plot 2, the antioxidant capacities of the AH160, AH160-Fe, and ASCG groups were higher (*p* < 0.05) than that of the control group (in the *in vitro* fraction and total antioxidant capacity). In the *in vitro* fermentation fraction, only the antioxidant capacity of the control-Fe group was significantly (*p* < 0.05) lower than that of the control group. The ANOVA analysis (Table 2) for plot 1 showed that the antioxidant capacity of the control-Fe group was significantly lower than those of the AH160 and ASCG-Fe groups (*p* < 0.05). In addition, the ASCG-Fe group had a higher antioxidant capacity than the ASCG group (*p* < 0.05). For plot 2, in addition to the aforementioned differences with respect to the control group, AH160 cucumbers had a higher antioxidant capacity compared with the other groups (*p* < 0.05), except for the ASCG group (no differences were found). In addition, the antioxidant capacity of the ASCG group was higher (*p* < 0.05) than those of the AH160-Fe, ASCG-Fe, and control-Fe groups.

#### Differences in the Antioxidant Capacities of the *In Vitro* Digestion–Fermentation Fractions

**Figure 3:**
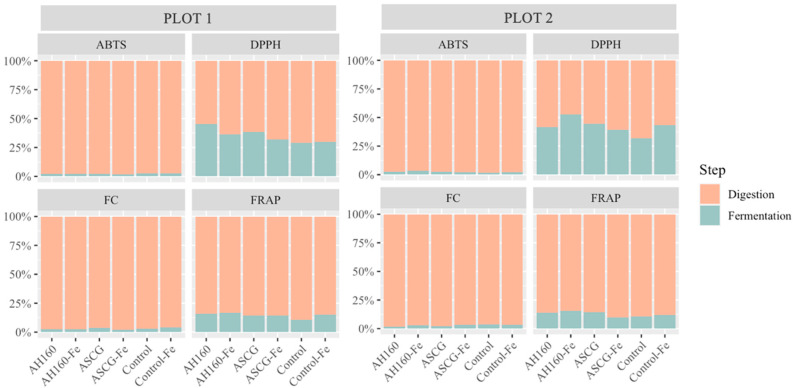
Contribution of each fraction to the total antioxidant capacity (ABTS, DPPH, FC, and FRAP) for all groups of cucumbers in both plots.

Figure 3 depicts the antioxidant capacity of both fractions obtained after *in vitro* digestion and fermentation. In general, the antioxidant capacity after *in vitro* fermentation decreased considerably compared with that obtained after *in vitro* digestion. This occurred for both ABTS and FC methods and in both plots. For the FRAP method, the *in vitro* fermentation fraction did not reach 25% for any of the treated groups of cucumbers or any of the plots. In the case of the DPPH method, it was observed that the contribution of the *in vitro* fermentation fraction to the total antioxidant capacity was higher than for the other methods, although it did not reach 50% in any of the treated cucumber groups in plot 1; in comparison, the AH160-Fe group exceeded 50% in plot 2. Therefore, *in vitro* digestion contributes more to the release of total antioxidant capacity in both plots.

### 3.3. Short-Chain Fatty Acids Produced after In Vitro Fermentation

**Figure 4:**
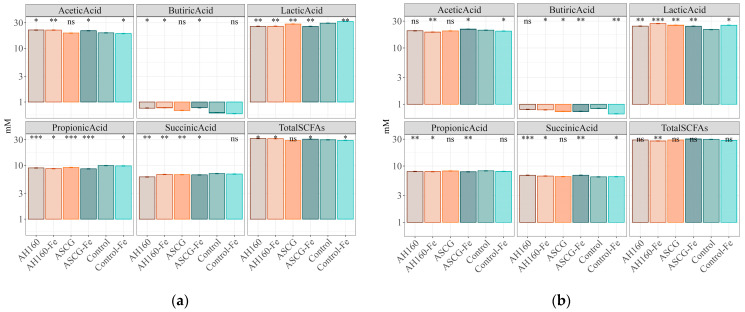
SCFAs produced after *in vitro* fermentation of cucumbers. (**a**) Plot 1. (**b**) Plot 2. Results are log10 transformed to improve visualization. Statistical analysis was performed using Student’s *t*-test with the control group as a reference. Statistical labels: *: *p* < 0.05, **: *p* < 0.01, ***: *p* < 0.001, ns: not significant.

SCFAs are health-promoting metabolites produced by gut microbes that feed on undigested nutrients, such as dietary fiber [14,29]. The SCFAs produced after *in vitro* fermentation of cucumbers from all groups were measured (See Supplemental Table S2). First, comparisons were made between the cucumber groups and the control group, and then comparisons were made among all groups. The results of plot 1 (Figure 4a) showed that, when taking into account the production of acetic acid, all cucumber groups were statistically significantly different (*p* < 0.05) from the control group, except the ASCG group, for which no differences were found. For butyric acid, higher levels of acetic acid were produced after fermentation of AH160, AH160-Fe, and ASCG-Fe cucumbers (*p* < 0.05) compared with the control group. For lactic and propionic acids, significant differences (*p* < 0.05) were found in the production of these short-chain fatty acids in all groups of cucumbers compared with the control group. The same was true for succinic acid, although in this case, the control-Fe group did not show statistically significant differences compared with the control group. Finally, with regard to the production of total SCFAs, statistically significant differences (*p* < 0.05) were found for all cucumber groups compared with the control group, except for the ASCG group.

When multiple comparisons (ANOVA) were made among all groups (Table 3) for plot 1, numerous statistically significant differences were found, in addition to those already mentioned with respect to the control. Of note were the differences found (*p* < 0.05) between the production of all SCFAs in the control-Fe group compared with AH160, AH160-Fe, and ASCG-Fe. It is also noteworthy to mention that differences (*p* < 0.05) in the production of short-chain fatty acids after the fermentation of ASCG cucumbers were found in almost all SCFAs compared with those of the AH160, AH160-Fe, ASCG, and control-Fe groups.

With regard to the results of plot 2 (Figure 4b), taking into account the production of acetic acid, all groups were statistically significantly different (*p* < 0.05) from the control group cucumbers, except for the AH160 and ASCG groups (no differences found). Butyric acid was produced at higher levels in control cucumbers compared with the other groups (*p* < 0.05), except for the AH160 cucumbers. As for lactic acid production, statistically significant differences (*p* < 0.05) were found in all cucumber groups with respect to the control group. The results for propionic acid production showed significant differences (*p* < 0.05) in all cucumber groups with respect to the control group, except the control-Fe and ASCG groups. Similar results were obtained for succinic acid, although in this case, the control-Fe group did show statistically significant differences from the control group. Finally, with regard to the total production of SCFAs, statistically significant differences (*p* < 0.05) were found only between the AH160-Fe group and the control group.

When the ANOVA analysis was carried out for plot 2 (Table 3), the levels of all short-chain fatty acids (except propionic acid) in the control-Fe group were different from those of AH160, AH160-Fe, and ASCG-Fe (*p* < 0.05). The fermentation of ASCG cucumbers gave rise to significantly (*p* < 0.05) different production of all SCFAs (except butyric acid between ASCG and ASCG-Fe) compared with the AH160-Fe and ASCG-Fe groups. Finally, the differences between the ASCG-Fe and AH160-Fe groups were also remarkable; in this case, it was found that the production of all short-chain fatty acids, except propionic acid, was different (*p* < 0.05) in both groups. See Supplemental Figure S1.

## 4. Discussion

This research has assessed whether spent coffee grounds, a type of biowaste with a toxic potential for plants that also contributes to CO_2_ release into the atmosphere [3,5], can be used after proper modification to improve the nutritional value of vegetables by means of agronomic biofortification with Fe. In general, the biochelates that induced the greatest increases in the Fe contents of cucumbers were the activated and functionalized hydrochar from SCG in both plots (Figure 1a). To better understand the dynamics of iron in this greenhouse experiment, the iron utilization efficiency (UE) for cucumbers was calculated in relation to the total amount of added iron. The following order was obtained in plot 1: AH160 (8.22) > AH160-Fe (0.148) > ASCG-Fe (0.032) > Control-Fe (0.0095) > ASCG (−13.41). In the case of plot 2, the results for this parameter were as follows: ASCG (4.61) > AH160-Fe (0.0508) > SCGA-Fe (−0.0078) > Control-Fe (−0.0747) > AH160 (−25.97). If we compare these results with those of [10], who used activated and functionalized Fe hydrochar to grow lettuces, similar trends were obtained. The activated and functionalized hydrochar at 160 °C had a higher UE than the activated and functionalized ASCG-Fe. This is a positive outcome when comparing the biochelates with the control-Fe cucumbers. In fact, the Fe contained in the hydrochar particles [10] could be released into the medium over time and have a residual effect in subsequent crop cycles [30]. In contrast, the commercial chelate (control-Fe) had a lower utilization efficiency, which can be explained by leaching of Fe over the cultivation period. The soil moisture was maintained at a potential of −20 kPa (which is higher than field capacity, −33 kPa), indicating the presence of excess water and a continuous leaching process. According to Weil and Brady [31], Fe is a micronutrient that is less available under conditions of high soil leaching.

The aim of agronomic biofortification is to improve the nutritional value of selected cultivars, in our case, cucumbers enriched with Fe. Thus, once it was demonstrated that SCG could be a source of biochelates with biofortification activity, the amount of Fe provided to the diet by each group of cucumbers was studied and compared with the European Fe reference intake for middle-aged men [28]. The AH160-Fe group (Table 4) was the cucumber group with the highest contribution of iron per serving (0.18 mg for plot 1 and 0.16 mg for plot 2) when compared with reference intakes. In contrast, the ASCG group provided the lowest iron levels to the diet per intake and per serving in plot 1, and the AH160 group in plot 2, which is consistent with the utilization efficiency data presented previously. The contribution of iron provided by the cucumbers, taking into account their daily intake in Spain, was very low (from 0.05 to 0.09% of daily requirements). The percentages of the contributions to daily intake and per serving were higher (from 1.40 to 2.29% of daily iron requirements) although still low, as most of the iron in the diet comes from foods of animal origin [32].

Cucumbers are vegetable foods, so they can contribute to the daily intake of bioactive compounds such as polyphenols [33]. According to a previous study [27], the daily intake of phenolic compounds in Spain is 1171 mg gallic acid equivalent/person/day (assessed using the Folin–Ciocalteu assay). Of these, approximately 80% come from fruits, vegetables, and cereals. In our study, Table 4 shows that the group of cucumbers from plots 1 and 2 with the lowest contribution to the daily intake of polyphenols was the control-Fe group. Previous research [32,34] found that iron can decrease the bioavailability of polyphenols and thus the antioxidant capacity of polyphenols during digestion.

The cucumbers with the highest contribution to the daily intake of phenolic compounds were the ASCG-Fe (for plot 1) and AH160 groups (for plot 2), reaching 660 and 822 mg/gallic acid/serving (Table 4). The richness in bioactive compounds of ASCG is recognized in the literature, as is their impressive biological effect, which is why they have several possible applications in the pharmaceutical, cosmetic, and food industries, among others [35]. Previous authors found that biochar can increase the concentration of polyphenols with high nutritional value in the short term [36], whereas in the long term, they can have the opposite effect. When the percentage contribution to the daily intake of polyphenols was studied, it ranged from 1.20 to 2.81% when the mean daily consumption of cucumbers in Spain was taken into account. However, if a more realistic approach is used (the intake of a serving), then the contribution reaches up to 70.2% (Table 4). It is important to note that in addition to phenolic species, other compounds can interact with the Folin–Ciocalteu reagent. Therefore, the total phenolic content may have been overestimated. The Folin–Ciocalteu assay should be seen as a measure of total antioxidant capacity rather than phenolic content. As phenolics are the most abundant antioxidants in most plants, in most cases the Folin–Ciocalteau assay gives a rough approximation of the total phenolic content [37].

In the case of antioxidant capacity, it is important to highlight that, in general, the groups of cucumbers containing iron (control-Fe, AH160-Fe) had lower antioxidant capacities than those groups with the same treatment but without iron (control and AH160). This was different for the ASCG-Fe and ASCG groups, with the non-iron group having higher antioxidant capacity in both plots. However, in the FC method, the opposite was observed for the samples from plot 2, with the ASCG group having higher amounts of polyphenols than ASCG-Fe. Once again, the influence on the quantity of total polyphenols in the presence of iron [35] could be a potential explanation for such behavior. Another important fact to be mentioned is the higher antioxidant capacity released during digestion compared with that released after fermentation. This could be related to the high digestibility of cucumber, as most of the compounds with antioxidant capacity were released after *in vitro* digestion, thereby not being available for metabolization by the gut microbiota in the colon.

An important aspect to take into account is the soil structure, which in this study had a direct impact on the nutritional properties of the cucumbers (See Supplemental Figure S2). The uptake of water and nutrients by plants can be limited by inadequate contact with the solid and liquid phases of the soil [38]. This could explain why such differences in soil could have a direct impact on the nutritional properties of cucumbers.

## 5. Conclusions

This work demonstrated that spent coffee grounds can be re-used as a source of smart biochelates to improve iron levels in cucumbers. The addition of activated hydrochar from SCG enriched with iron (AH160-Fe) to plants produced the highest increase in Fe levels in cucumbers for both plots, with the AH160-Fe group being the highest contributor to iron intake per serving compared with the other groups; however, in general, the iron contribution of cucumbers to the human diet was low. Cucumbers grown with activated hydrochar from SCG (AH160) or activated spent coffee grounds (ASCG) demonstrated the highest contribution to the daily intake of polyphenols. In terms of antioxidant capacity, an inverse relationship was found between the presence of iron, as the groups grown with iron biochelates had lower antioxidant capacities compared with non-iron groups; however, this relationship was not consistent across all groups studied. Differences in SCFA production were observed among the groups of cucumbers, showing that the soil structure (which differed in both plots) may influence the chemical composition of the samples. Both the iron contents and chemical compositions of cucumbers may vary significantly depending on the growing conditions and presence of iron, which in turn may affect the contribution of these cucumbers to the dietary intake of nutrients and antioxidants; this may have important implications for human health and nutrition. For future research, growing conditions (such as soil structure and composition) should be taken into account, as they greatly affect the nutritional content of Dutch cucumbers. Another aspect to be considered is the evaluation of sustainability, carrying out life cycle and sustainability assessments to determine the environmental and economic impact of using iron biochelates derived from spent coffee grounds in agriculture. This could include evaluating the environmental costs and benefits compared with conventional methods of iron supplementation in agriculture, as well as evaluating the long-term economic viability of this practice.

## Figures and Tables

**Table 1:** Treatment details of the greenhouse experiment.

Nº	Treatment	Description	Fe Added (mg/kg)
1	Control	No bioproduct	0
2	Control-Fe	Commercial chelate (EDDHA-Fe, 6%)	10
3	ASCG	Activated spent coffee grounds (SCG)	0.104
4	AH160	Activated hydrochar obtained from SCG at 160 °C	0.105
5	ASCG-Fe	Activated SCG functionalized with Fe	21
6	AH160-Fe	Activated hydrochar obtained from SCG at 160 °C functionalized with Fe	23

**Table 2:** Differences in Fe contents and antioxidant capacity (FRAP, DPPH, ABTS, and FC assays) between cucumber groups. Differences in the two plots were studied separately. Statistical analysis was performed using ANOVA and Tukey’s test. Statistical labels: *: *p* < 0.05, ns: not significant.

	Fe Content	FRAP Assay	DPPH Assay	ABTS Assay	FC Assay
Plot 1					
AH160-Fe/AH160	*	ns	ns	ns	ns
ASCG/AH160	*	ns	ns	ns	ns
ASCG-Fe/AH160	ns	ns	ns	*	ns
Control/AH160	*	ns	ns	*	ns
Control-Fe/AH160	*	ns	ns	ns	ns
ASCG/AH160-Fe	*	ns	ns	ns	ns
ASCG-Fe/AH160-Fe	*	ns	ns	ns	ns
Control/AH160-Fe	ns	ns	ns	ns	ns
Control-Fe/AH160-Fe	*	ns	ns	ns	*
ASCG-Fe/ASCG	*	ns	ns	*	*
Control/ASCG	*	ns	ns	ns	ns
Control-Fe/ASCG	*	ns	ns	ns	ns
Control/ASCG-Fe	ns	ns	ns	*	ns
Control-Fe/ASCG-Fe	ns	ns	ns	*	*
Control-Fe/Control	ns	ns	ns	ns	ns
Plot 2					
AH160-Fe/AH160	*	ns	ns	ns	*
ASCG/AH160	*	ns	ns	ns	ns
ASCG-Fe/AH160	*	ns	ns	*	*
Control/AH160	*	ns	ns	*	*
Control-Fe/AH160	*	ns	*	ns	*
ASCG/AH160-Fe	*	ns	ns	ns	*
ASCG-Fe/AH160-Fe	*	ns	*	*	ns
Control/AH160-Fe	*	ns	ns	ns	*
Control-Fe/AH160-Fe	*	ns	ns	ns	ns
ASCG-Fe/ASCG	*	ns	*	ns	*
Control/ASCG	*	ns	*	*	*
Control-Fe/ASCG	*	ns	ns	ns	*
Control/ASCG-Fe	ns	ns	ns	*	ns
Control-Fe/ASCG-Fe	ns	ns	*	ns	ns
Control-Fe/Control	*	ns	*	*	ns

**Table 3:** Differences in short-chain fatty acid production among cucumber groups. Differences in the two plots were studied separately. Statistical analysis was performed using ANOVA and Tukey’s test. Statistical labels: *: *p* < 0.05, ns: not significant.

Plot 1	Acetic Acid	Butyric Acid	Lactic Acid	Propionic Acid	Succinic Acid	Total SCFAs
AH160-Fe/AH160	*	ns	ns	*	*	ns
ASCG/AH160	ns	*	*	*	*	*
ASCG-Fe/AH160	*	ns	ns	*	*	*
Control/AH160	*	*	*	*	*	*
Control-Fe/AH160	*	*	*	*	*	*
ASCG/AH160-Fe	*	*	*	*	ns	*
ASCG-Fe/AH160-Fe	ns	ns	ns	ns	*	*
Control/AH160-Fe	*	*	*	*	*	*
Control-Fe/AH160-Fe	*	*	*	*	*	*
ASCG-Fe/ASCG	*	*	*	*	ns	*
Control/ASCG	ns	ns	*	*	*	ns
Control-Fe/ASCG	*	*	*	*	*	ns
Control/ASCG-Fe	*	*	*	*	*	*
Control-Fe/ASCG-Fe	*	*	*	*	*	*
Control-Fe/Control	*	ns	*	*	ns	*
Plot 2	Acetic acid	Butyric acid	Lactic acid	Propionic acid	Succinic acid	Total SCFAs
AH160-Fe/AH160	*	ns	*	ns	*	*
ASCG/AH160	ns	*	*	*	*	ns
ASCG-Fe/AH160	*	*	ns	ns	ns	*
Control/AH160	ns	ns	*	*	*	*
Control-Fe/AH160	*	*	*	ns	*	*
ASCG/AH160-Fe	*	*	*	*	*	*
ASCG-Fe/AH160-Fe	*	*	*	ns	*	*
Control/AH160-Fe	*	*	*	*	*	*
Control-Fe/AH160-Fe	*	*	*	ns	*	*
ASCG-Fe/ASCG	*	ns	*	*	*	*
Control/ASCG	ns	*	ns	ns	ns	ns
Control-Fe/ASCG	ns	*	ns	*	ns	*
Control/ASCG-Fe	*	*	*	*	*	ns
Control-Fe/ASCG-Fe	*	*	*	ns	*	*
Control-Fe/Control	*	*	ns	*	*	ns

**Table 4:** Contribution of cucumber consumption to daily antioxidant capacity and Fe intake in the Spanish diet.

Group	Analytical Assay	Fe/Daily Intake * (mg Fe/Day)		Fe/Serving Intake ^†^ (mg Fe/Serving)		Mean Contribution to Daily Fe Intake Compared with Previous Data ^#^ (%)		Mean Contribution to Daily Fe Per Serving Intake ^#^ (%)	
		Plot 1	Plot 2	Plot 1	Plot 2	Plot 1	Plot 2	Plot 1	Plot 2
Control	Fe content	0.01	0.01	0.13	0.15	0.07	0.07	1.66	1.84
Control-Fe	Fe content	0.01	0.01	0.13	0.14	0.07	0.07	1.68	1.70
ASCG	Fe content	0.00	0.01	0.11	0.15	0.06	0.08	1.40	1.93
AH160	Fe content	0.01	0.00	0.15	0.11	0.07	0.05	1.82	1.33
ASCG-Fe	Fe content	0.01	0.01	0.14	0.14	0.07	0.07	1.78	1.81
AH160-FE	Fe content	0.01	0.01	0.18	0.16	0.09	0.08	2.29	2.06
Group	Analytical assay	Polyphenols/Daily Intake * (mg Gallic Acid/Day)		Polyphenols/Serving Intake ^†^ (mg Gallic Acid/Serving)		Mean Contribution to Daily Polyphenol Intake Compared with Previous Data ^$^ (%)		Mean Contribution to Daily Polyphenol Intake per Serving ^$^ (%)	
		Plot 1	Plot 2	Plot 1	Plot 2	Plot 1	Plot 2	Plot 1	Plot 2
Control	FC assay	21.1	16.3	529	407	1.81	1.39	45.1	34.7
Control-Fe	FC assay	16.0	14.0	401	351	1.37	1.20	34.2	29.9
ASCG	FC assay	16.8	29.9	421	748	1.44	2.56	35.9	63.9
AH160	FC assay	21.8	32.9	546	822	1.87	2.81	46.6	70.2
ASCG-Fe	FC assay	26.4	17.3	660	434	2.25	1.48	56.3	37.0
AH160-Fe	FC assay	23.8	19.6	596	489	2.04	1.67	50.9	41.8

* Based on the daily consumption of the Spanish population. ^†^ Based on the intake from one serving. ^#^ Based on dietary reference intakes [28]. ^$^ Based on previous data [27].

## Data Availability

Raw data are available upon request to Prof. Rufián-Henares due to privacy.

## Supplementary Materials

The following supporting information can be downloaded at: https://www.mdpi.com/article/10.3390/antiox13040465/s1, Figure S1: Principal component analysis (PCA) of short chain fatty acids produced after in vitro fermentation, depending on treatment (AH160, ASCG, control) and Fe biofortification; Figure S2: Principal component analysis (PCA) biplot chart. Total antioxidant capacity (FRAP, DPPH, ABTS and FC), short chain fatty acids production and Fe content of the samples were taken into account. Arrows indicate in which plot the variable influence is higher; Table S1: Fe content (mg/100g fresh weight) and total antioxidant capacity of different groups of cucumbers, expressed as mmol Trolox/Kg cucumber fresh weight for FRAP, DDPH and ABTS assays. For FC assay total antioxidant capacity is expressed as mg equivalent gallic acid/Kg cucumber; Table S2: Short chain fatty acids content (expressed in mM) of different groups of cucumbers.

## Abbreviations

SCG: Spent Coffee Grounds; TEAC: Trolox Equivalent Antioxidant Capacity; GAE: Gallic Acid Equivalents; FC: Folin–Ciocalteu; SCFAs: Short-Chain Fatty Acids.
