# Supplementary material for: Biochelates from Spent Coffee Grounds Increases Iron Levels in Dutch Cucumbers but Affects Their Antioxidant Capacity
Source: Antioxidants (Basel). 2024 Apr 15;13(4):465. doi: 10.3390/antiox13040465 (PMC11047731; doi:10.3390/antiox13040465)
Supplement: Supplementary file 1 [file antioxidants-13-00465-s001.zip › antioxidants-2944759-supplementary.pdf]

Biochelates from spent coffee grounds increases iron levels of Dutch cucumbers but affects their antioxidant capacity

SUPPLEMENTAL INFORMATION

Supplemental Table 1. Fe content (mg/100g fresh weight) and total antioxidant capacity of different groups of cucumbers, expressed as mmol Trolox/Kg cucumber fresh weight for FRAP, DDPH and ABTS assays. For FC assay total antioxidant capacity is expressed as mg equivalent gallic acid/Kg cucumber.

**SAMPLE**					
**PLOT 1**	**Fe content**	**FRAP assay**	**DPPH assay**	**ABTS assay**	**FC assay**
Control	0.09 ± 0.00	4.24 ± 0.66	1.49 ± 0.92	57.3 ± 0.24	3524 ± 733
Control-Fe	0.09 ± 0.00	3.24 ± 1.13	1.65 ± 0.22	55.3 ± 0.89	2673 ± 115
ASCG	0.07 ± 0.00	2.60 ± 0.05	1.24 ± 0.03	62.7 ± 8.69	2806 ± 199
ASCG-Fe	0.10 ± 0.00	3.17 ± 0.44	1.96 ± 0.67	87.5 ± 0.26	4398 ± 131
AH160	0.10 ± 0.00	2.66 ±0.03	1.27 ± 0.19	59.4 ± 0.42	3640 ± 109
AH160-Fe	0.12 ± 0.00	2.89 ± 0.11	1.37 ± 0.31	73.5 ± 11.0	3974 ± 69.0
**PLOT 2**	**Fe content**	**FRAP assay**	**DPPH assay**	**ABTS assay**	**FC assay**
Control	0.10 ± 0.00	4.17 ±1.66	1.88 ± 1.33	85.8 ± 1.29	2711 ± 100
Control-Fe	0.09 ± 0.00	2.69 ± 0.08	1.06 ± 0.04	56.6 ± 2.31	2338 ± 258
ASCG	0.10 ± 0.00	2.86 ± 0.35	1.30 ± 0.09	57.1 ± 0.39	4989 ± 29.1
ASCG-Fe	0.10 ± 0.00	4.54 ± 0.23	1.69 ± 0.11	71.2 ± 1.92	2890 ± 336
AH160	0.07 ± 0.00	3.07 ± 0.01	1.51 ± 0.05	52.4 ± 1.10	5483 ± 415
AH160-Fe	0.11 ± 0.00	2.79 ± 0.02	1.23 ± 0.02	44.5 ± 10.8	3261 ± 95.8

Supplemental Table 2. Short chain fatty acids content (expressed in mM) of different groups of cucumbers.

SAMPLE						
**PLOT 1**	**Lactic acid**	**Acetic acid**	**Succinic acid**	**Propionic acid**	**Butiric acid**	**Total SCFAs**
Control	29.4 ± 0.06	19.4 ± 0.00	7.06 ± 0.01	9.91 ± 0.02	0.63 ± 0.01	29.9 ± 0.01
Control-Fe	31.7 ± 0.08	18.8 ± 0.05	6.91 ± 0.03	9.78 ± 0.01	0.61 ± 0.01	29.2 ± 0.07
ASCG	28.4 ± 0.04	19.1 ± 0.15	6.74 ± 0.01	9.12 ± 0.01	0.70 ± 0.01	29.0 ± 0.13
ASCG-Fe	25.7 ± 0.00	21.1 ± 0.05	6.67 ± 0.03	8.63 ± 0.02	0.78 ± 0.00	30.7 ± 0.04
AH160	25.7 ± 0.02	21.8 ± 0.12	6.11 ± 0.02	8.95 ± 0.02	0.77 ± 0.00	31.5 ± 0.14
AH160-Fe	25.8 ± 0.00	21.8 ± 0.03	6.80 ± 0.01	8.68 ± 0.06	0.78 ± 0.01	31.3 ±0.10
**PLOT 2**	**Lactic acid**	**Acetic acid**	**Succinic acid**	**Propionic acid**	**Butiric acid**	**Total SCFAs**
Control	21.2 ± 0.10	20.7 ± 0.11	6.42 ± 0.01	8.23 ± 0.02	0.84 ± 0.01	29.7 ± 0.13
Control-Fe	25.3 ± 0.01	19.9 ± 0.05	6.47 ± 0.00	8.01 ± 0.05	0.68 ± 0.01	28.6 ± 0.00
ASCG	25.3 ± 0.21	20.1 ± 0.03	6.49 ± 0.04	8.17 ± 0.03	0.75 ± 0.01	29.0 ± 0.04
ASCG-Fe	24.3 ± 0.06	21.6 ± 0.17	6.86 ± 0.01	7.91± 0.02	0.75 ± 0.01	30.2 ±0.18
AH160	24.4 ± 0.05	20.2 ± 0.02	6.85 ± 0.00	8.00 ± 0.01	0.81 ± 0.00	29.0 ± 0.01
AH160-Fe	27.1 ± 0.13	19.1 ± 0.07	6.63 ± 0.00	7.94 ± 0.03	0.80 ± 0.00	27.9 ± 0.10

Supplemental Figure 1: Principal component analysis (PCA) of short chain fatty acids produced after *in vitro* fermentation, depending on treatment (AH160, ASCG, control) and Fe biofortification.

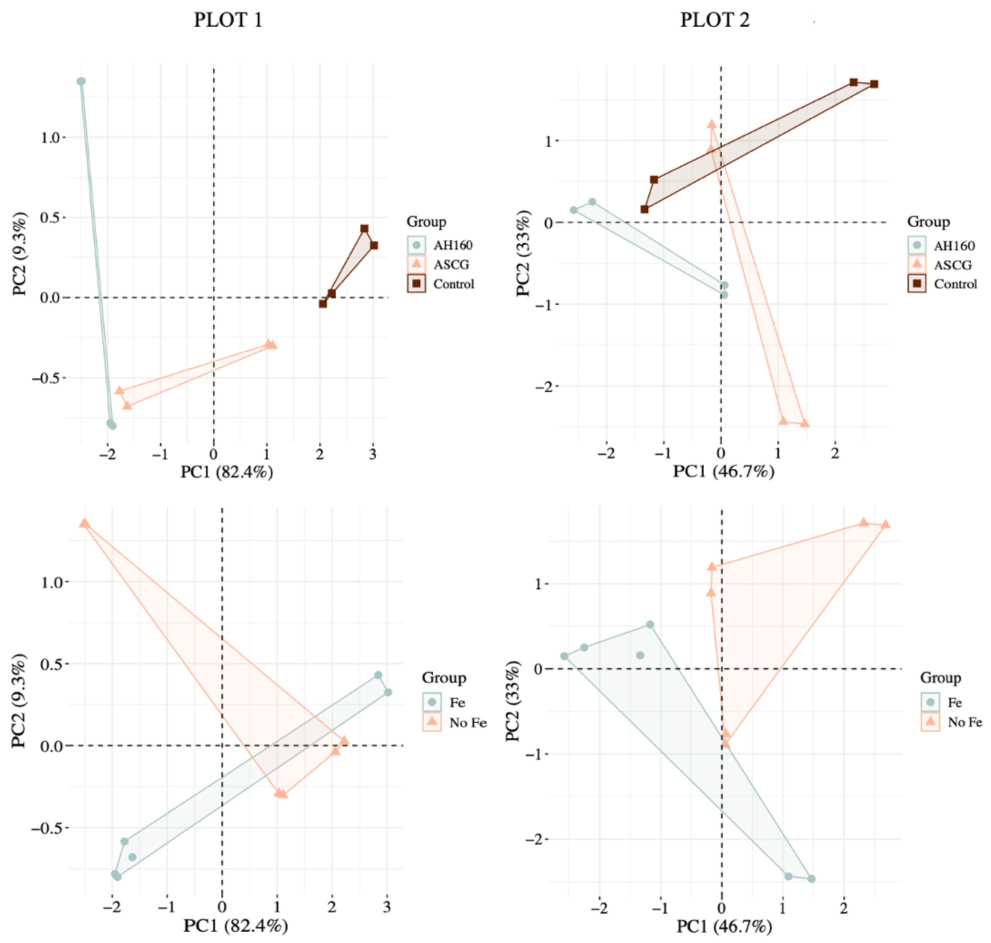

The differences found in SCFAs production were numerous in both plots. Figure 5 depicts four principal component analyses (PCA) carried out with the data for all SCFAs. In the case of plot 1, PC1 explained an 82.4% of the variability, while PC2 explained an additional 9.3%. Overall, samples were more clustered depending on the treatment applied to the soil (AH160, ASCG, control) than depending on iron biofortification. On the contrary, for plot 2 samples clustered better depending on the iron fortification. For this plot, the first component explained a 46.7% while the second accounted for another 33%.

Supplemental Figure 2: Principal component analysis (PCA) biplot chart. Total antioxidant capacity (FRAP, DPPH, ABTS and FC), short chain fatty acids production and Fe content of the samples were taken into account. Arrows indicate in which plot the variable influence is higher.

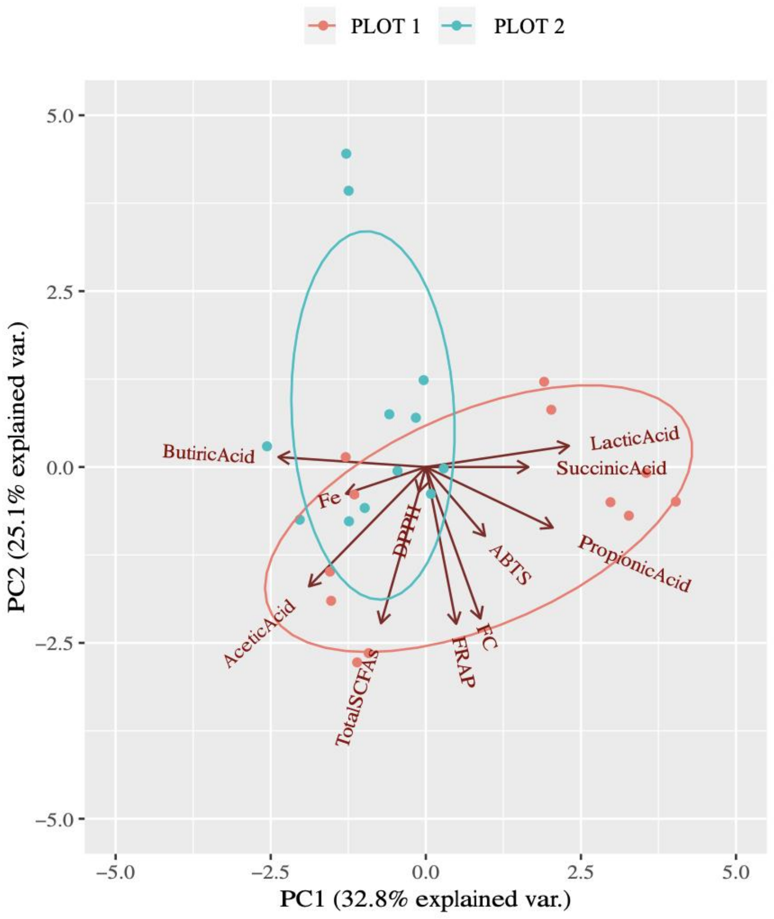

In order to study the significance of the different variables studied in both plots, a principal component analysis was performed (Figure 6). Since the variables included in the analysis where very different (Fe content, antioxidant capacity and SCFAs) the variability explained for both plots was not very high (total 57.9%, 32.8 for PC1 and 25.1 for PC2). The samples from plot 1 showed higher contributions to the explained variability by lactic, succinic and propionic acids, ABTS, FC and FRAP. In the case of plot 2, butyric acid was the highest

contributor. It is well known that soil structure affects plant growth in many ways. The uptake of water and nutrients by plants can be limited by inadequate contact with the solid and liquid phases of the soil. This could explain why such differences in soil could have a direct impact on the nutritional properties of cucumbers.
